# P02.132. Repeatability of pulse diagnosis in traditional Indian Ayurveda medicine

**DOI:** 10.1186/1472-6882-12-S1-P188

**Published:** 2012-06-12

**Authors:** V Kurande, R Waagepetersen, E Toft

**Affiliations:** 1 Aalborg University, Aalborg, Denmark; 2 Department of Mathematical Sciences, Aalborg University, Aalborg, Denmark

## Purpose

In Ayurveda, pulse diagnosis is the unique diagnostic method that determines the proportion of diagnostic variables (vata, pitta and kapha); however, this is only justifiable if pulse diagnosis yields a consistent result. Though pulse diagnosis has a long historical use, still there is lack of quantitative measures on e.g., reliability of the diagnostic method. Reliability means consistency of information. Consistent diagnosis leads to consistent treatment and is important for clinical practice, education and research. The objective of this study is to study methodology to test the test-retest reliability (repeatability) of pulse diagnosis. Another objective is to provide additional interpretation of Cohen’s weighted kappa statistic for analysis of categorical pulse diagnosis variables.

## Methods

A double-blinded, controlled, observational clinical trial was conducted at the Art of Living Center in Copenhagen, Denmark. The same doctor, an expert in Ayurvedic pulse diagnosis, examined the pulse of 17 healthy subjects twice in a random order without seeing them. For statistical analysis, a distance measure on pulse diagnosis variables was developed. Cohen's weighted kappa statistic was used as a measure of intra-rater reliability. Permutation tests were used to test the hypothesis of homogeneous diagnosis.

## Results

The hypothesis of homogeneous diagnosis was rejected on the 5% significance level (p-value of 0.02). According to the Landis and Koch scale, a weighted kappa value of 0.42 for pulse diagnosis corresponds to a ‘moderate’ level of agreement.

## Conclusion

Results show that there was a reasonable level of consistency between two pulse diagnoses. Further studies using the developed methodology are required to quantify inter-subject and intra-subject agreement for greater understanding of reliability of pulse diagnosis. The developed statistical methodology appears to be appropriate for assessing reliability of pulse diagnosis and will be beneficial for further studies of pulse diagnosis, tongue diagnosis and body constitution (prakriti) assessment.

